# Transcriptome Analysis Reveals Putative Target Genes of *APETALA3-3* During Early Floral Development in *Nigella damascena* L.

**DOI:** 10.3389/fpls.2021.660803

**Published:** 2021-06-04

**Authors:** Yves Deveaux, Natalia Conde e Silva, Domenica Manicacci, Martine Le Guilloux, Véronique Brunaud, Harry Belcram, Johann Joets, Ludivine Soubigou-Taconnat, Etienne Delannoy, Hélène Corti, Sandrine Balzergue, Jose Caius, Sophie Nadot, Catherine Damerval

**Affiliations:** ^1^Université Paris-Saclay, INRAE, CNRS, AgroParisTech, Génétique Quantitative et Evolution-Le Moulon, Gif-sur-Yvette, France; ^2^Université Paris-Saclay, CNRS, INRAE, Univ Evry, Institute of Plant Sciences Paris-Saclay (IPS2), Orsay, France; ^3^Université de Paris, CNRS, INRAE, Institute of Plant Sciences Paris-Saclay (IPS2), Orsay, France; ^4^Univ Angers, Institut Agro, INRAE, IRHS, SFR QUASAV, Angers, France; ^5^Université Paris-Saclay, CNRS, AgroParisTech, Ecologie Systématique Evolution, Orsay, France

**Keywords:** APETALA3, RNA-seq, AP3 target genes, petal, *Nigella damascena*, floral mutant, Ranunculaceae, *Arabidopsis*

## Abstract

Even though petals are homoplastic structures, their identity consistently involves genes of the APETALA3 (AP3) lineage. However, the extent to which the networks downstream of AP3 are conserved in species with petals of different evolutionary origins is unknown. In Ranunculaceae, the specificity of the AP3-III lineage offers a great opportunity to identify the petal gene regulatory network in a comparative framework. Using a transcriptomic approach, we investigated putative target genes of the AP3-III ortholog NdAP3-3 in *Nigella damascena* at early developmental stages when petal identity is determined, and we compared our data with that from selected eudicot species. We generated a *de novo* reference transcriptome to carry out a differential gene expression analysis between the wild-type and mutant *NdAP3-3* genotypes differing by the presence vs. absence of petals at early stages of floral development. Among the 1,620 genes that were significantly differentially expressed between the two genotypes, functional annotation suggested a large involvement of nuclear activities, including regulation of transcription, and enrichment in processes linked to cell proliferation. Comparing with *Arabidopsis* data, we found that highly conserved genes between the two species are enriched in homologs of direct targets of the AtAP3 protein. Integrating AP3-3 binding site data from another Ranunculaceae species, *Aquilegia coerulea*, allowed us to identify a set of 18 putative target genes that were conserved between the three species. Our results suggest that, despite the independent evolutionary origin of petals in core eudicots and Ranunculaceae, a small conserved set of genes determines petal identity and early development in these taxa.

## Introduction

Petals are floral organs that play a major role in pollinator attraction. In flowers with a differentiated perianth, petals are defined as the second whorl of sterile organs surrounding the androecium; the first whorl of sterile organs is composed of sepals, which protect the fertile organs during development. A recent reconstruction of the ancestral angiosperm flower suggests that the ancestral flower has an undifferentiated perianth and floral organs inserted in whorls or along a spiral (Sauquet et al., 2017). Perianth differentiation has evolved several times independently, and consequently, the term “petal” is not indicative of organ homology across angiosperms (Irish, 2009; Ronse de Craene and Brockington, 2013).

The analysis of floral mutants in two core eudicot species, *Antirrhinum majus* and *Arabidopsis thaliana*, has been a turning point for understanding the genetic control of floral organ identity by proposing a model based on four main functions: A, B, C, and E. The A-function specifies sepals, A‐ and B-functions determine petals, B‐ and C-functions specify stamens, and C-function specifies carpels (Coen and Meyerowitz, 1991). The E-function combines with the three other functions to specify floral identity (Pelaz et al., 2001). Most genes involved in A-, B-, C-, and E-functions, encode transcription factors belonging to the MIKC-type MADS-box gene family (reviewed in Theißen and Gramzow, 2016). They are known to act in tetrameric protein complexes (“floral quartets”; Theißen et al., 2016; Yan et al., 2016). In particular, B-function relies on two proteins APETALA3/DEFICIENS (AP3) and PISTILLATA/GLOBOSA (PI) in *Arabidopsis*/*Antirrhinum*, respectively, which function as an obligate heterodimer within tetrameric complexes (Goto and Meyerowitz, 1994).

*AP3* and *PI* belong to two paralogous gene lineages that result from an ancient duplication preceding the angiosperm radiation. Additional duplications took place in the *AP3* and *PI* lineages during angiosperm evolution, at different points in time (Lee and Irish, 2011). In the *AP3* lineage, an ancient duplication event occurred at the base of the core eudicots, resulting in the TM6 and euAP3 gene lineages. The euAP3 lineage is characterized by a C-terminal motif that differs from the paleoAP3 motif present in TM6 and other AP3 lineages (Kramer et al., 1998, 2006). EuAP3 genes are thought to play an important role in the formation and diversification of petals in core eudicots (Irish, 2009). Independently, two successive duplications of AP3 have occurred at the base of the order Ranunculales, the sister group to all other eudicots, giving rise to three paralogous lineages: AP3-I, AP3-II, and AP3-III (Rasmussen et al., 2009). The *PI* lineage evolution is also complex and dynamic with cases of multiple recent duplications (Zahn et al., 2005). In Ranunculales, the B-function involves generally the three AP3 paralogous and at least one PI paralogous genes. Heterodimerization of PI proteins with AP3 proteins of the different lineages has been observed in several species (Kramer et al., 2007; Wang et al., 2016; Galimba et al., 2018). While *PI* gene expression is essential for both stamen and petal identity (Kramer et al., 2007; Wang et al., 2016), the function of the different paralogous AP3 genes with regard to stamen and petal identity has diverged (Kramer et al., 2007; Sharma and Kramer, 2013; Wang et al., 2016). In particular, it has been shown that the presence of petals is strongly correlated with the expression of AP3-III orthologs (Kramer et al., 2003; Di Stilio et al., 2005; Rasmussen et al., 2009), and that the absence of petals in some species is linked to a disruption of the *AP3-3* locus (Zhang et al., 2013). In two Ranunculaceae species where functional studies can be conducted using Virus-Induced Gene Silencing (VIGS), namely, *Aquilegia coerulea* and *Nigella damascena*, it has been shown that expression of *AP3-3* genes is required for petal identity (Sharma et al., 2011; Gonçalves et al., 2013; Wang et al., 2016). These lines of evidence indicate that AP3-3 orthologs play a crucial role in determining petal identity in Ranunculaceae.

Petals, with their multiple independent origins, have been consistently associated with the expression of B-function genes (Ambrose et al., 2000; Drea et al., 2007; Mondragón-Palomino and Theissen, 2008). Therefore, they are a good case study of the conservation of gene networks downstream of B-function proteins. While *Arabidopsis AP3* gene is involved in both petal and stamen development, the petal specificity of the Ranunculaceae *AP3-3* genes offers a good opportunity to unravel a petal-specific gene regulatory network. The target genes of MADS-box proteins are beginning to be identified in model species, mainly in *Arabidopsis* (Bey et al., 2004; Kaufmann et al., 2009, 2010; Wuest et al., 2012; Ó’Maoiléidigh et al., 2013; Pajoro et al., 2014). High throughput sequencing methods now open the way for comparing possible target genes in a wider range of non-model species representing diverse organ morphologies and evolutionary origins (Thomson et al., 2017).

Expression of B-function genes is required at every stage of development to obtain full organ identity. In *N. damascena*, we observed that the presence and morphology of petals differed according to the timing of inactivation of *NdAP3-3* by VIGS (Gonçalves et al., 2013). Early inactivation results in the absence of petal formation, resulting in a phenocopy of the spontaneous mutant lacking petals known since the 16th century (Toxopéus, 1927). When inactivation takes place at later stages, a range of intermediate morphologies between petals and sepals are observed. In this paper we intended to characterize the early target genes of NdAP3-3 that determine the full petal identity. Because of the large size of the *N. damascena* genome (~10 Gb), no genomic data are available for this species yet. To circumvent this limiting factor, we assembled and annotated a floral reference transcriptome and characterized differential gene expression between wild-type and mutant genotypes at the *NdAP3-3* locus differing by the presence vs. absence of petals. The wild type vs. mutant comparison gives the unique opportunity to investigate the early stages of floral development when petals are initiated or not. Therefore, differentially expressed genes are candidates for direct or indirect targets of the NdAP3-3 protein involved in petal identity and early development. Genes were annotated and compared to known or suspected targets of B-function genes in other eudicot species (Bey et al., 2004; Wuest et al., 2012; Jiang et al., 2020), and the conservation of the petal gene network is discussed.

## Materials and Methods

### Plant Material and Tissue Collection

Segregating populations were obtained by selfing plants issued from two commercial seed lots obtained from horticultural companies (Royal Fleur and Truffaut) as described in Gonçalves et al. (2013). The heterozygosity level of these mother plants is unknown. For analysis of the early stages, the progeny of F2 plants (F3 offspring), which were homozygous either for the wild-type *P* allele (P morph, with petals) or the mutant *p* allele (T morph, without petals), were sown in three replicates. Each of the three replicates consisted of a mix of eight seeds from the same six *PP* or *pp* F2 mother plants. Plants were grown in pots in randomized blocks in a growth chamber under controlled conditions (18 h day at 25°C, 6 h night at 16°C). Another set of *PP* plants was grown independently to collect late developmental stages. The genotype at the *NdAP3-3* locus was checked for all plants by PCR using primers flanking the MITE element insertion site in intron 2, which is a marker for the *p* allele (Gonçalves et al., 2013).

Floral buds were collected at different stages, based on the developmental schedule previously described by Jabbour et al. (2015). Stage 1 corresponds to 1 day after floral transition marked by the beginning of stem extension; at this stage, only sepals are initiated in both genotypes. Stage 2 occurs 3 days later, and corresponded to petal initiation (*PP* plants) or internal sepal initiation (*pp* plants). Approximately 20 terminal flower meristems were dissected under a stereomicroscope allowing stage inspection for each condition (morph × stage × replicate). Flower buds were collected from 32 *PP* plants at three late stages (bud diameter 5–6 mm, 6–8 mm, and just before anthesis), and the organs were dissected and kept separately (sepals, petals, stamens, and carpels). All dissected floral tissues were immediately frozen in liquid nitrogen and stored at −80°C until RNA extraction.

### RNA Extraction and RNA-Sequence Analysis

Total RNA from floral meristems and dissected organs was extracted using the RNeasy Plant Mini Kit (Qiagen) with the additional DNAse I step according to the manufacturer’s instructions. Total RNA from each tissue was checked for integrity on an RNA_Nano chip, using an Agilent 2,100 bioanalyzer (Agilent Technologies, Waldbroon, Germany). Twelve libraries were constructed for the two morphs at the two earliest developmental stages in three replicates. Two additional libraries were constructed from the dissected floral organs of the P morph, the first one by pooling equal amounts of RNA from the three non-petal organs at the three late developmental stages, and the second one by pooling RNA from petals only at these three stages. The libraries were constructed with the TruSeq-stranded mRNA library Prep kit (Illumina, California, USA) with 300-bp size for libraries from early-stage material and 260-bp size for dissected material. Libraries were paired-end (PE) sequenced with a read length of 150 bp for early-stage material and 100 bp for dissected material using an Illumina HiSeq2000 at the Genoscope Laboratory (Evry, France). Lane distribution and barcoding gave approximately 20–30 million PE reads per sample. For each sample, raw data (fastq) were trimmed with Trimmomatic (Bolger et al., 2014) with a Phred Quality Score Qscore > 20, and read lengths > 30 bases. Ribosome sequences were removed with the sortMeRNA tool (Kopylova et al., 2012).

All steps of the study, from growth conditions to bioinformatic analyses, were managed in a CATdb database (Gagnot et al., 2007, http://tools.ips2.u-psud.fr/CATdb/) with ProjectID NGS2013_09_AAP-IDEEV and NGS2015_16_Ranunculaceae. This project was submitted from CATdb to the international public repository GEO (Gene Expression Omnibus, Edgar et al., 2002, http://www.ncbi.nlm.nih.gov/geo, GSE159429).

### Reference Transcriptome Assembly

Assembly of the reference transcriptome was made using Trinity (version 2.4, Grabherr et al., 2011). To balance between the different genotypes, developmental stages, and organs, we combined the following six samples for a total of ~130 million oriented PE reads: four libraries were from floral buds of the P and T morphs at stages 1 and 2, and two libraries were from petal and non-petal tissues from the P morph at later stages. Trinity was run with default parameters and a kmer size of 32. Contigs that were smaller than 200 bases were removed, and 81,194 contigs were finally obtained. iAssembler (version 1.3, Zheng et al., 2011) was then used for scaffolding contigs and reducing redundancy, with 97% identity for sequence clustering. For simplicity, hereafter we use the term transcript or gene to refer to the contigs, which may include paralogs, alleles, and alternative splicing of the same gene.

### Functional Annotation and Homology Search

Before annotating the transcriptome, we predicted all coding sequences. First, the TransDecoder software (v5.0.2; Perina et al., 2016; Roy et al., 2018) was used to predict all putative open reading frames (ORFs) among assembled transcript sequences (default parameters). These predicted ORFs were then searched for Pfam domain hits (v28) using the HMMER software (3.1b2, default settings; Eddy and Pearson, 2011). Final coding region predictions were achieved by running TransDecoder a second time taking into account the Pfam domain search output. Functional annotations were performed by searching for sequence similarity and protein domains. Similarity of the predicted proteins with sequences from the UniRef90 (Suzek et al., 2015) and TAIR databases (Huala et al., 2001; Rhee et al., 2003) was determined using Blast (ncbi-blast-2.6.0+, E-value < 1.10–3; Altschul et al., 1990). InterProScan software (5.29–68.0; Zdobnov and Apweiler, 2001; Jones et al., 2014) was used to search for InterPro domain hits (default settings). We extracted Gene Ontology (GO) and GO slim terms (functions, locations, and biological roles) from Blast and InterProScan outputs with a lab script (Ashburner et al., 2000; The Gene Ontology Consortium, 2001).

BlastP tool was used to compare *N. damascena* predicted proteins to the 43,550 proteins of *A. coerulea* from Phytozome (322-v3.1). We kept only the best hit for each *Nigella* protein (E-value ≤ 0.01). Blastn was also used to search for the homology of some unannotated transcripts in the *A. coerulea* genome.

Nine transcripts with differential expression between morphs and without functional annotation were chosen randomly to check whether they could correspond to novel potentially *Nigella*-specific sequences or were misassembled transcripts. Primers were designed based on the full sequences of these nine transcripts and used to amplify from cDNA and/or gDNA (Supplementary Table S1).

### Differential Gene Expression Analysis

Reads corresponding to the three replicates of stages 1 and stage 2 floral buds of the P and T morphs were mapped on the reference transcriptome. Every PE read in each sample was mapped against the complete list of contigs with Bowtie2 (Langmead and Salzberg, 2012, local option). Ninety six percent of the reads could be mapped, demonstrating the quality of transcriptome assembly. All PE reads with multi-hits, meaning mapped on sequence regions shared by isoforms, were removed. We kept only PE reads associated with a unique contig, ensuring specific quantification of gene expression. The method generated quantitative data for 70,491 contigs.

Low-expression genes were filtered to include only contigs (genes) that have at least 1 read count per million (CPM, counts per million) after normalization, in at least two samples. 34,614 transcripts (that include *NdAP3-3*) were thus retained for quantitative analysis. The coefficients of determination calculated on normalized counts between pairs of replicates for each stage–organ combination were high (R2 > 0.95). To assess how the overall variability is structured among the 12 datasets (2 morphs × 2 stages × 3 replicates), a principal component analysis was carried out on the normalized counts for these 34,614 informative transcripts using the FactoMineR package. Using the EdgeR package v3.8.6 (Robinson et al., 2010), we then fitted the normalized count data to a model that considered the effect of plant morph (2 levels), developmental stage (2 levels), replicate (3 levels), as well as the morph by stage and morph by replicate interactions. We performed contrasts to (i) compare the P and T morphs at stage 1, at stage 2, and at both stages on average; (ii) compare the developmental stages in the P morph, the T morph, and in both morphs on average; and (iii) test the morph by stage interaction. In each case, *p*-values for statistical significance were adjusted following the Benjamini and Hochberg’s (1995) FDR method.

To quantify the level of differential expression between morphs, we calculated the log2 ratio of the mean of the normalized counts of T over P for the three replicates (hereafter M_LFC) at stage 1, stage 2, or both together. A heatmap of the log2-normalized counts of the two morphs at the two developmental stages in the three replicates was drawn with the heatmap function in R.

As a complementary approach to identify possible targets of the NdAP3-3 transcription factor, we correlated the expression of the remaining 34,613 transcripts for quantitative analysis with the expression of the *NdAP3-3* gene in the P morph. Because the effect of the developmental stage was high for *NdAP3-3* and for most transcripts, we normalized the data per stage. The Pearson’s correlation coefficient was then calculated for each of the 34,613 transcripts and the corresponding FDR was calculated. Although this analysis may be of very low power (normalization and correlation based on 6 data points – three replicates for each developmental stage) and some statistical hypotheses may not be fulfilled (normality of expression data), we used it to strengthen the evidence of a functional interaction between differentially expressed genes and NdAP3-3. We chose to focus on genes with a Pearson’s correlation coefficient ≥ 0.8, which corresponded to an FDR value ≤ 0.26.

Functional category enrichment/depletion among differentially expressed vs. non-differentially expressed genes were tested using Chi-squared contingency tests (*p* < 0.001).

### Consistency Between RNA-Sequence Quantitative Data and Quantitative RT-PCR Results

A set of 15 genes were randomly selected for comparison of quantitative expression measured by RNAseq-normalized counts and quantitative RT-PCR. The RNA pools from the three replicates used for RNA-seq were used to synthesize cDNAs for qRT-PCR. Primer pairs were designed from the transcript sequences (Supplementary Table S1) and validated by qRT-PCR on a dilution series. Four genes were initially chosen as possible references, based on their stable expression within the range of expression of the 15 genes of interest. After qRT-PCR analysis, we finally selected RN20658, a pentatricopeptide repeat (PPR)-containing protein as the best reference. Relative expression was calculated using the 2^−ΔΔCt^ method.

### Identification of Conserved Petal Genes and Homologs of Direct Target Genes of AP3 in *Nigella damascena*

First, we searched the *N. damascena* annotated transcriptome for homologs of the *A. thaliana* genes (AGI-ID) whose expression is correlated with that of MADS-box genes of the ABCE model (Supplementary Table S4). This was done using the Expression Angler program and the AtGenExpress Plus ‐ Extended tissue Compendium data set (Toufighi et al., 2005), as previously described (Damerval et al., 2019).

Second, we focused on genes involved in petal formation in *Arabidopsis* and searched for their homologs in the *Nigella* transcriptome. These genes are often members of gene families and functional diversification may be species specific. Therefore, for each gene of the *Nigella* transcriptome, we sorted the 10 best AGI-ID hits to identify homology with any member of *Arabidopsis* petal gene families. The obtained dataset was then filtered using two different AGI-ID lists to identify *Nigella* homologs of *Arabidopsis* genes that were (i) co-expressed with *AtAP3*, and/or (ii) differentially expressed between the wild type and the *ap3.3* mutant in flowers. The first AGI-ID list was composed of the 100 best positively and 100 best negatively correlated genes to *AtAP3* in *Arabidopsis* (Toufighi et al., 2005), in addition to *AtPI* and *AtAP3* (AtAP3-BCG). The second AGI-ID list contained differentially expressed genes between wild type and mutant regardless of whether they are considered in the literature to be putative direct targets of AtAP3 or not (AtDEG, Mara and Irish, 2008; Wuest et al., 2012). Whereas the first dataset aimed to identify genes associated with petal and stamen development in a broad sense, the second dataset was more specific to the AtAP3 downstream network. Both lists were then used to identify the *Nigella* genes that were homologous, respectively, to the AtAP3-BCG (NdHBCG) and the AtDEG (NdH). Finally, these genes were characterized as (i) being differentially expressed between the two *Nigella* morphs (NdHBCG-DEG and NdH-DEG, respectively) and (ii) having as best homolog, an AtAP3 direct target gene or a paralog of an AtAP3 direct target gene.

Third, to identify additional putative target genes in the set of genes differentially expressed in *Nigella*, we took advantage of the ChIP-sequence analysis of the whole genome of *A. coerulea* for the detection of AP3-3 protein (AcAP3-3) target sequences that was conducted at a late floral developmental stage (Jiang et al., 2020).

## Results

### Reference Transcriptome Assembly and Functional Annotation

The floral reference transcriptome of *N. damascena* was assembled *de novo* from a pool of transcripts obtained from early developmental stages when organs are initiated to later stages when all organs are formed and are growing (see Material and Methods). 71,319 contigs with a mean length of 1,160 bp and an N50 length of 1843 bp were generated. These parameters were in the same range as those of another *N. damascena* transcriptome elaborated from floral buds, bracts, and leaves (95,758 unigenes, mean length 933 bp, N50 of 1711 bp, Zhang et al., 2020). As expected, compared to the data from the annotated whole genome of another Ranunculaceae species, *A. coerulea* (43,550 transcripts, mean gene length; 1755 bp, N50 length: 2133 bp, https://phytozome.jgi.doe.go), the contigs of our *Nigella* transcriptome were shorter and more numerous. Two factors may account for this difference: our material was heterozygous, and classical assembly tools tend to fall short of complete gene coverage and generate too many smaller than expected contigs.

In the absence of a fully sequenced and annotated genome of *N. damascena*, a total of 53,133 peptides were predicted from 35,409 different transcripts. InterProScan identified 19,347 transcripts encoding peptides with at least one protein domain and a GO annotation. A BLAST search of UniRef90 allowed 5,603 genes to be annotated against 5,751 proteins. Selecting the GO:0003700 (DNA-binding transcription factor activity), we found 355 genes with at least one domain corresponding to 20 types of transcription factors (Supplementary Figure S1). Among these, the K-box, AP2/ERF, and basic leucine zipper were the most represented (20, 19, and 15%, respectively). Homologs of all ABCE MADS-box floral genes were found, except for *AP1*.

**Figure 1:**
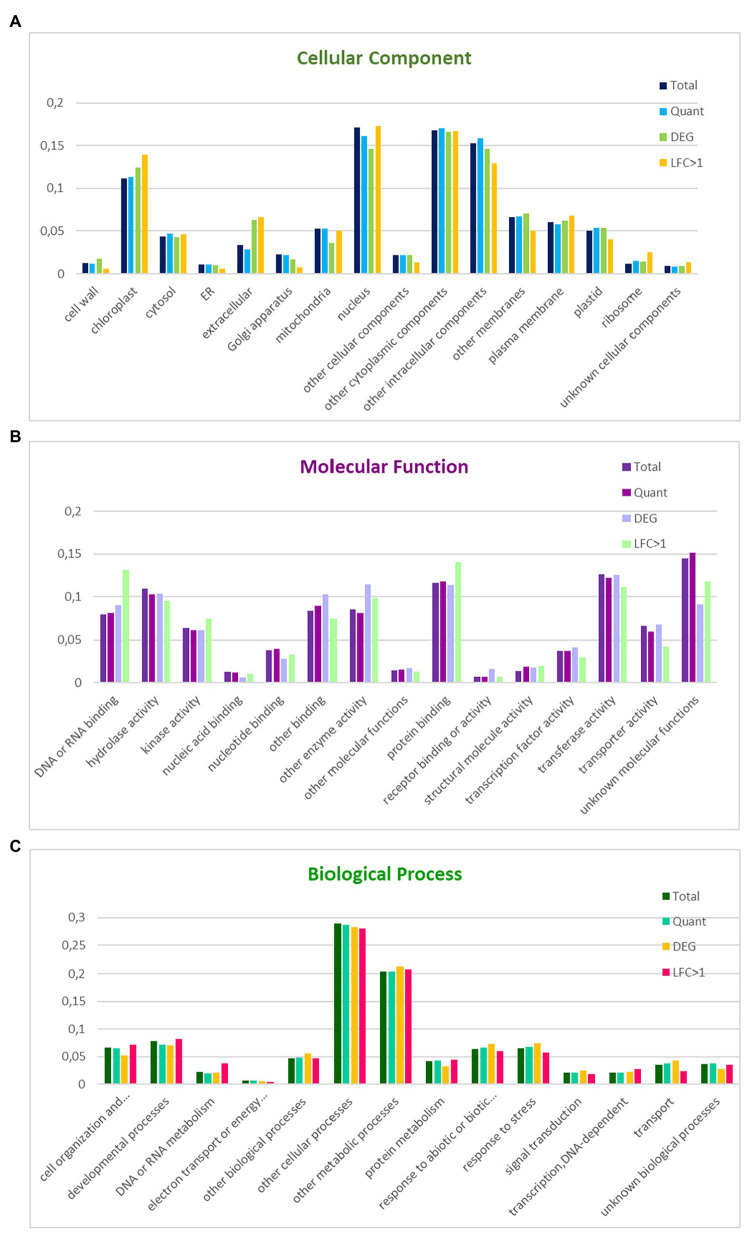
Distribution of GO slim terms in the floral reference transcriptome, the set of informative transcripts used in the quantitative analysis, and the genes differentially expressed between floral morphs in *Nigella damascena*. Distributions are compared within GO cellular component **(A)**, molecular function **(B)**, and biological process **(C)**. Total: reference transcriptome; Quant: transcripts in quantitative analysis; DEG: genes differentially expressed between the two morphs; LFC > 1: most differentially expressed genes between the two morphs.

A high percentage of coding regions could be annotated against *Arabidopsis* (29,877–84%) and *A. coerulea* (30,700–87%) proteins. The GO slim annotation of the *N. damascena* transcriptome was based on homology with *Arabidopsis* genes annotated with *Arabidopsis* GO slim terms (see Material and Methods). The two best-represented categories within biological process were “other cellular processes” (29%) and “other metabolic processes” (20%); within cellular components, the four best-represented categories were “nucleus” (17%), “other cytoplasmic components” (17%), “other intracellular components” (15%), and “chloroplast” (11%); among molecular functions, the best-represented categories were “protein binding” (12%), “hydrolase activity” (11%), and “transferase activity” (13%; Figure 1).

### Analysis of Differential Gene Expression During Petal Initiation in the Two *Nigella damascena* Floral Morphs

Quantitative RNA-seq was conducted at two early flower developmental stages framing petal initiation, as defined in Jabbour et al. (2015). We compared gene expression in two pools of full-sib F3 genotypes homozygous for the *NdAP3-3* alleles determining the presence (*PP* genotype, P morph) or absence (*pp* genotype, T morph) of petals. Genes that are expressed differentially between the two morphs should therefore include genes from the petal initiation network downstream of *NdAP3-3*, but also a limited number of genes in close linkage disequilibrium with *NdAP3-3* that are expressed at these floral developmental stages.

Based on informative read counts, 34,614 transcripts, which include *NdAP3-3*, were included in the quantitative analysis (Supplementary Table S2). We tested the consistency of gene expression patterns observed with RNA-seq counts using qRT-PCR and by randomly choosing a few genes and replicates. Among a set of 14 randomly chosen transcripts and the *NdAP3-3* gene, the expression patterns were similar between the two methods for *NdAP3-3* and 11 of the 14 genes (Supplementary Figure S2).

**Figure 2:**
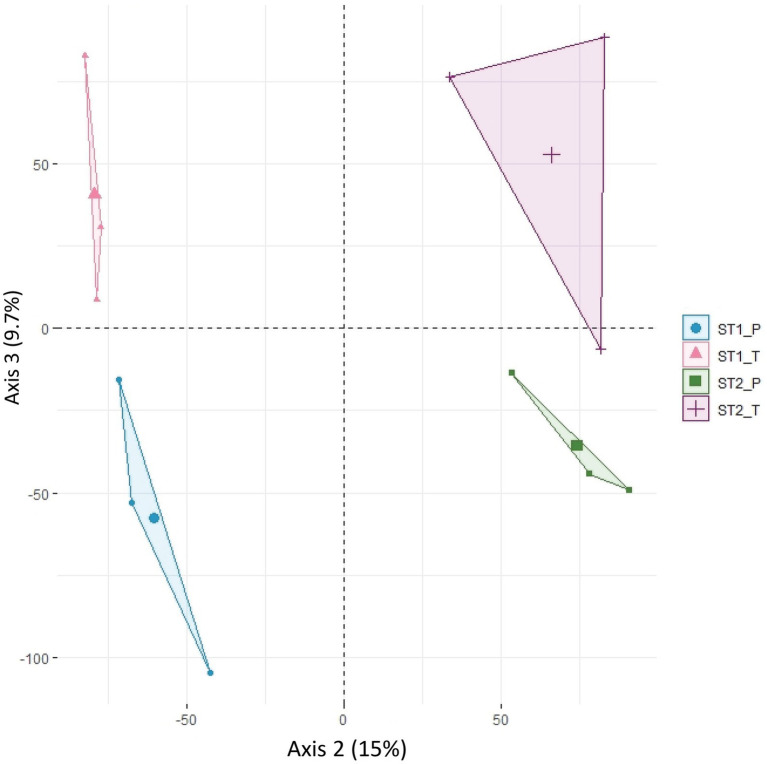
Principal component analysis (PCA) of normalized counts for 34,614 informative transcripts. PCA was done on the four-stage x morph combinations and the three biological replicates. Axis 2 (15% of the variability) separates individuals according to developmental stage (stage 1 vs. stage 2) and axis 3 (9.7% of the variability) separates them according to morph (P morph vs. T morph). ST1_P, ST1_T: individuals at stage 1 of P and T morph, respectively; ST2_P, ST2_T: individuals at stage 2 of P and T morph, respectively.

To assess how variability is structured among the 12 (2 morphs x 2 stages x 3 replicates) datasets, a principal component analysis (PCA) was carried out on the expression of the 34,614 genes. The variation in expression between replicates accounted for 31% of the overall variability, while the second axis of the PCA separated developmental stages 1 and 2 (15% of the variability) and the third axis separated the two morphs (9.7%; Figure 2; Supplementary Figure S3). The relatively high part of variability between replicates may be accounted for by the residual heterozygosity in the F3 generation, even though we tried to homogenize the background by using pools of plants. The developmental stage was the major factor for differential expression. 4,959 genes were differentially expressed (DEGs) between stages 1 and 2 (FDR < 0.05) in at least one of the two genotypes; about half of these (2,133, with twice as many genes showing an increased expression in stage 2 compared with stage 1 than vice versa) were differentially expressed in both genotypes, suggesting that they participate in a general “flower” or “sepal” developmental program but not in petal specification.

To investigate the possible direct or indirect targets of NdAP3-3, we focused on the 1,620 genes that were found to be differentially expressed between morphs at either stage or over both stages on average (FDR < 0.05, Supplementary Table S3; Supplementary Figure S4). This DEG set between morphs was highly enriched in genes also exhibiting a stage effect (652 genes, 40% vs. 14% of all genes analyzed). The number of DEGs between the two morphs was higher at stage 2 (884 genes) than at stage 1 (580 genes), which is consistent with petal initiation at stage 2 in the P morph, while at stage 2 the T morph produces additional sepal-like primordia. Interestingly, a significant morph by stage interaction was found for 74 transcripts (FDR < 0.05), most of which also exhibited a significant morph effect at one or both stages (63 out of 74). Among the B-function genes, only the three *NdAP3* paralogs were found differentially expressed between the two morphs, *NdAP3-1* (RN021161) at stage 2 but not stage 1, *NdAP3-2* (RN002991) at stage 1 but not stage 2, and *NdAP3-3* (RN035793) at both stages, as expected.

**Figure 3:**
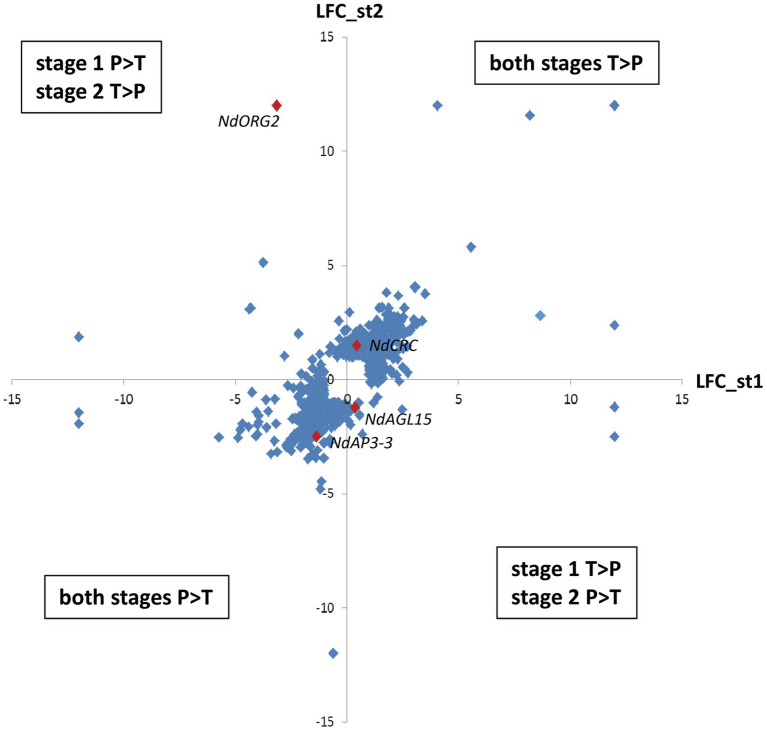
Differential expression between morphs at the two developmental stages for the most differentially expressed genes. Differential expression is expressed by Log2fold change, and only genes with |M_LFC| > 1 at one or both developmental stages are represented. The y-axis represents differential expression between the two morphs at stage 2 and the x-axis represents differential expression at stage 1. The upper right quadrant includes genes more expressed in the T morph at both stages, while the lower left quadrant includes genes more expressed in the P morph at both stages. The two other quadrant group genes that have reversed differential expression between the two morphs at the two stages. Examples of genes illustrating these situations are represented by red diamonds. Genes not expressed at one stage have been arbitrarily attributed M_LFC = +12: (T morph) or −12: (P morph).

Among these 1,620 DEGs, 739 exhibited at least two-fold difference in expression level between morphs (|M_LFC| > 1) at one or both developmental stages. The most represented GO term within the 243 annotated genes in this set was “nucleus” (GO:0005634, 74 genes), including DNA binding (GO:0003677), protein binding (GO:0005515), and regulation of transcription activity (GO:0006355). Other than NdAP3-3 (RN035793), homologs of transcription factors, such as Crabs Claw (NdCRC, RN058291); Reduced Vernalization Response 1 (NdVRN1, RN059576); OBP3-responsive gene 2 (NdORG2 with two isoforms, RN057454 and RN012090), and Agamous-like 15 (NdAGL15, RN008012), as well as histones and proteins involved in the maintenance of cell division (RN047478 and RN003149) and several F-Box proteins were found (Supplementary Table S3). Differential expression between morphs generally showed the same trend at both stages: 400 genes were more expressed in the T morph than in the P morph (e.g., *NdCRC*), while 275 genes were more expressed in the P morph than in the T morph (e.g., *NdAP3-3*, RN047478 and RN003149) at both developmental stages (Supplementary Table S3; Figure 3). For 64 transcripts, including homologs of ORG2 genes, the difference in expression between the two morphs was reversed between the two developmental stages (e.g., P > T at stage 1 and T > P at stage 2, Figure 3).

To identify the petal gene network in *Nigella*, we searched for the DEGs that were most correlated with wild-type *NdAP3-3* gene expression. We calculated the correlation between the expression of the 34,613 transcripts and the expression of *NdAP3-3* in the P morph, then focused on the correlation values among the set of 739 transcripts with a |M_LFC| > 1. We found 94 transcripts with a Pearson’s correlation coefficient ≥ 0.8, which corresponded to an FDR of 26%. Among these, about one-third could be annotated against the *Arabidopsis* proteome. The four most correlated transcripts (|r| > 0.99, FDR < 0.01) were unannotated and likely non-coding (Supplementary Table S3).

Forty-nine percent of the differentially expressed genes could not be annotated (792 out of 1,620, Supplementary Table S3), mostly because they did not include a coding phase longer than 100 amino acids. This proportion appears significantly less than the proportion in the whole set of quantitatively analyzed transcripts (57%, *p* < 0.00001). The alignment of the unannotated transcripts against the genomic sequence of *A. coerulea* revealed a significant percentage of similarity for 90 transcripts. Additionally, we verified a set of nine transcripts by PCR amplification to check whether they could correspond to *N. damascena* sequences with unknown homology or misassembled transcripts. Three sequences could not be amplified and one gave a mix of two different sequences. For the remaining five sequences, the amplified fragments covered 80.8–89.6% of the total length and corresponded to the expected sequence, with a few single-nucleotide polymorphisms. Among these validated unannotated transcripts, one included a putative coding phase of at least 100 amino acids and four were likely to be non-coding.

### Functional Enrichment Analyses

18,883 of the 34,614 transcripts included in the quantitative analysis, including 771 DEGs between the two morphs and 243 genes with a |M_LFC| > 1 could be annotated with GO terms. The proportion of genes represented in the different GO terms was compared between the different gene sets and with the annotation of the whole transcriptome within each of the three major GO slim categories (Figure 1). Compared with genes that are not differentially expressed between morphs, the morph DEG set was enriched in the extracellular, cell wall and chloroplast components, other enzyme activities, and receptor binding or receptor activity, and was depleted in mitochondrial components, cell organization, and biogenesis and protein metabolism processes. Interestingly, among the morph DEGs, those with a |M_LFC| > 1 were enriched in ribosome components, DNA or RNA binding, cell organization and biogenesis, and/or DNA or RNA or protein metabolism, and were depleted in transport and response to stress processes (Figure 1).

### Identification of Conserved Floral Genes and Putative NdAP3-3 Target Genes Using a Comparative Approach

The shared expression patterns that result from functional constraints imposed by the developmental process and the cell microenvironment are good indicators of the conservation of floral gene regulatory networks (Davila-Velderrain et al., 2014). To determine the extent to which the gene network involved in floral organ development is conserved in eudicots, we searched for homologs of genes in *Nigella* with expression patterns that are best correlated (BCGs) with that of ABCE MADS-box genes in *A. thaliana*, as done previously in *Grevillea juniperina* (Damerval et al., 2019). Analysis of the annotated *N. damascena* transcriptome revealed a rate of conservation of BCGs that was identical to that observed in *G. juniperina* using the same approach (Supplementary Figure S5A). In addition, *N. damascena* and *G. juniperina* have 75% of their BCGs in common with an *r*-value ≥ 0.75 (Supplementary Table S5; Damerval et al., 2019). Homologs of all ABCE genes except *AP1* were found in *N. damascena*, and most of the genes that were highly correlated with them were also conserved between *Nigella* and *Arabidopsis* (Supplementary Table S5). Individual analyses of the 25 BCGs showed that on average, 49% of homologs of the ABCE MADS-box correlated genes were conserved. Homologs of *AP1* and *AP3* correlated genes were slightly over-represented, whereas homologs of *AG* and *SEP1/3* correlated genes were under-represented (Supplementary Figure S5B). Overall, good conservation of the floral genes is observed between the species.

**Figure 4:**
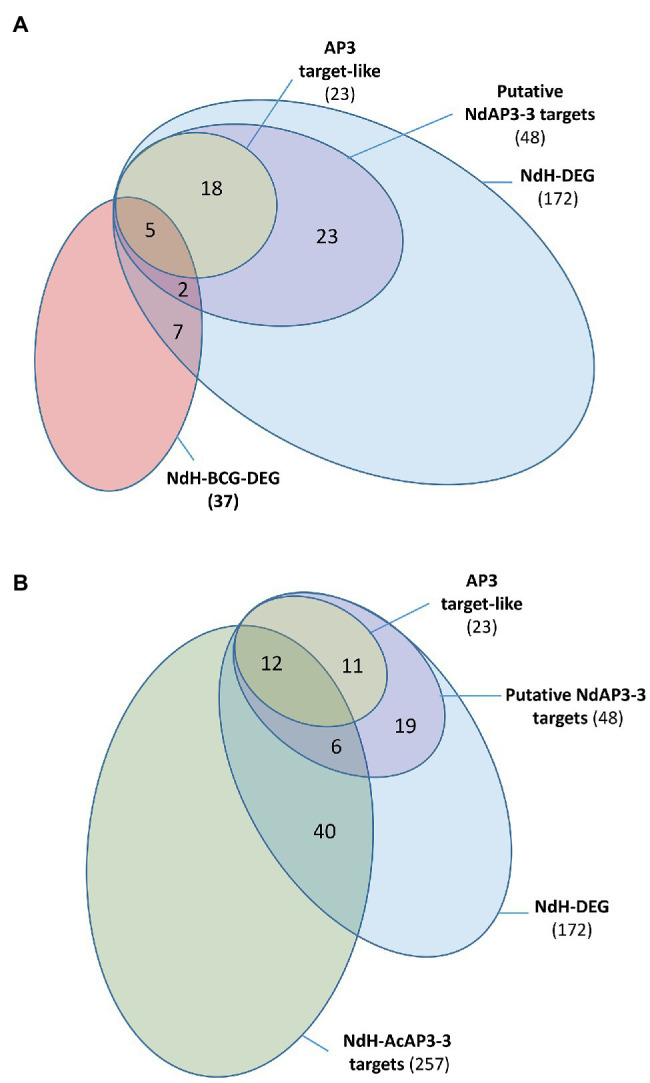
Venn diagram representing the distribution of the *Nigella* transcripts into subcategories. (**A**) NdH-DEG and NdH-BCG-DEG are *Nigella* genes which are differentially expressed between the two morphs and homologous to *Arabidopsis* genes which are differentially expressed between the wild type and the *ap3.3* mutant and the best correlated genes to *AtAP3*, respectively. Putative AP3-3 target genes include the best homologs of the AtAP3 target genes (AP3 target-like) and paralogs of AtAP3 target genes in *Nigella* (see details in Supplementary Table S6). (**B**) NdH-AcAP3-3 are the *Nigella* best homologs of the *Aquilegia* AcAP3-3 target genes (see details in Supplementary Table S8). The other categories are the same as panel **(A)**.

To be considered as a direct target of a transcription factor, a gene must have a binding signal in its regulatory regions and its expression must be regulated by this protein. In *Arabidopsis*, such a study combining genome-wide site detection and wild type vs. mutant comparison was conducted to identify the target genes of the floral transcription factor AtAP3 (Wuest et al., 2012). Because no genomic data are available in *Nigella*, we opted for a comparative approach, using the *Arabidopsis* data, to identify the putative target genes of NdAP3-3. We searched for homologous genes in *Arabidopsis* and *Nigella* whose expression is altered by the inactivation of their respective AP3 petal identity genes and identify those that are homologs to AtAP3 target genes. Only 20% of the 1,075 DEGs between the *Arabidopsis ap3.3* mutant and wild type (AtDEG, Supplementary Table S6A) were found to be direct targets of AtAP3 (Wuest et al., 2012). Among the 4,910 *Nigella* transcripts that are homologous to AtDEG (NdH), 172 were differentially expressed between the two *Nigella* floral morphs (NdH-DEG). Twenty-eight percent of the NdH-DEG were homologous to AtAP3 direct target genes (48 out 172), either as best hits to *Arabidopsis* target genes (23 out 172, 13%) or as a paralog of the target genes (25 out 172, 15%, Figure 4A).

The overlapping of the AtAP3-BCG set with that of the conserved DEG set between *Arabidopsis* and *Nigella* (NdH-DEG) was also analyzed to identify the core gene set of the petal development network. When considering the 202 *AtAP3* BCGs (AtAP3-BCG, Supplementary Table S6B), only 9% were differentially expressed between mutant and wild type (19 AtBCG-DEG), but almost half (9 out of 19, 47%) were classified as putative direct targets of the AtAP3 protein (Supplementary Table S6B). Among the 1,310 *Nigella* transcripts that were homologous to the AtAP3-BCG (NdH-BCG), ~3% (37) were differentially expressed between the two *Nigella* morphs (NdH-BCG-DEG). Within this group, the proportion of homologs of AtAP3 direct target genes was 13% (5 out 37) when considering only the best hits, and 18% (7 out 37) if paralogs of target genes are included (Figure 4A).

When restricting the analysis to the set of *Nigella* DEG genes that were the best hits of *Arabidopsis* AtBCG-DEGs and AtDEGs, the percentage of homologs of AtAP3-target genes increased to 45% (5 out of 11, Supplementary Table S6B) and 49% (23 out of 47, Supplementary Table S6A), respectively. Thus, the highly conserved gene set between the two species seems to be enriched in direct targets of AtAP3 protein. In addition, the expression of the majority of highly conserved homologs is affected in the same way in both the *Arabidopsis ap3.3* and *Nigella NdAP3-3* null mutants, as shown by the LFC in expression level during the early stages of flower development (Supplementary Table S7). This further suggests that these homologs of AtAP3 target genes could be putative direct targets of the NdAP3-3 protein in *N. damascena*. Among these putative conserved target genes, we found the *AP3* paralogs themselves, other transcription factor genes, such as *CRC*, *DRNL*, *AINTEGUMENTA-like 5*, and genes involved in cell wall biogenesis, gibberellin biosynthesis, and signal transduction (Supplementary Table S7).

Finally, to identify additional target genes in the set of genes differentially expressed between the two floral morphs in *Nigella*, we carried out a comparative analysis with the AP3-3 (AcAP3-3) target genes detected in the full genome of *A. coerulea*, another Ranunculaceae species (Jiang et al., 2020). Among the 1,620 DEGs found in *Nigella*, 257 were homologous to AcAP3-3 target genes (NdH-AcAP3-3; Figure 4B). When considering the set of 172 homologs that are differentially expressed in both *Arabidopsis* and *Nigella* (NdH-DEG), 18 (12+6) putative targets were found conserved between the three species, 30 (19+11) were specific to *Arabidopsis* and *Nigella*, and 239 were specific to *Nigella* and *Aquilegia* (Supplementary Table S8). Interestingly, among the target genes that were conserved between the three species, one-third of the *Nigella* genes homologous to a paralog of AtAP3-target genes were found to be the best homologs of an AcAP3-3 target gene (6 out of 18, Supplementary Table S8). Among the 239 putative conserved target genes between *Nigella* and *Aquilegia*, 40 genes were homologous to genes that were differentially expressed between the *Arabidopsis* wild type and *ap3.3* mutant although not direct targets of the AtAP3 protein.

## Discussion

Petals in Ranunculaceae are believed to have evolved independently of petals in core eudicots (Irish, 2009; Carrive et al., 2020). In both Ranunculaceae and core eudicots, however, B-function genes are instrumental for their identity and development. In Ranunculaceae, of the three AP3 paralogous lineages, the AP3-III orthologs have shown to be key genes for petal identity (Sharma et al., 2011; Gonçalves et al., 2013; Wang et al., 2016). In this study, we took advantage of the spontaneous *NdAP3-3* mutation in *N. damascena* that results in a floral morph without petals to characterize early target genes of NdAP3-3, which determine petal identity and first steps of development. Then we adopted a comparative perspective to address the question of the conservation of this early gene network, using the wealth of knowledge available in *Arabidopsis* as a reference point for core eudicots, and the recently available results obtained from developing petals in *A. coerulea* (Jiang et al., 2020).

### Representativeness of the *de novo* Floral Reference Transcriptome Illustrated by ABCE MADS-Box Genes

We found in our transcriptome all the MADS-box genes involved in the *N. damascena* ABCE model of floral organ identity (Wang et al., 2016). In *Arabidopsis*, *AP1* is one of the two genes involved in the A-function. In *N. damascena*, no *AP1* homolog was found, but several isoforms of the *NdAGL6* gene, which from functional analysis is suspected to be an A-function gene (Wang et al., 2016) were identified. The high proportion of homologs of the *Arabidopsis* AP1 best correlated genes in *Nigella* suggests that the A-function network is as represented as the networks of the other functions, even though it may have a different master control gene, possibly *NdAGL6*. The slight overrepresentation of homologs of AP1-BCGs, as well as of AP3-BCGs, could be due to a bias toward early developmental–stage genes and a preponderance of perianth developmental genes at the expense of reproductive organ genes in the *Nigella* transcriptome (Supplementary Figure S5B). This hypothesis could also account for the comparatively slight underrepresentation of homologs of *AG* and possibly *SEP1/3*-correlated genes.

### Effect of *NdAP3-3* Mutation on the Expression of Other B-Function Genes at Early Stages of Petal Development

In *Arabidopsis*, the B-function AtAP3 and AtPI proteins interact as a heterodimer to maintain their expression at a high level at late stages. However, their initial expressions are independent (Goto and Meyerowitz, 1994). In *N. damascena*, all the B-function genes are more or less expressed in petal depending on the developmental stage (Gonçalves et al., 2013; Wang et al., 2016). *NdPI1*, which encodes a protein shown to interact with NdAP3-3 in yeast two-hybrid (Wang et al., 2016), was not differentially expressed between morphs during early petal development; no differential expression was observed either for its paralog *NdPI2* (Supplementary Table S2). These data further support that their initial expression does not depend on AP3/PI protein–protein interaction, as shown in *Arabidopsis*. By contrast, *NdAP3-2* appeared significantly more expressed in the T morph than the P morph at stage 1, but the increase in expression between stages was higher in the P morph than in the T morph, resulting in no significant difference between morphs at stage 2. The reverse situation was observed for the *NdAP3-1* isoform with no differential expression between morphs at stage 1 but a greater increase in expression in the P morph than in the T morph resulting in a significant difference at stage 2. Thus, the differential expression profiles of *NdAP3-1* and *NdAP3-2* suggest either a regulatory control of their expression by NdAP3-3 (Wang et al., 2016; Jiang et al., 2020), or some kind of interaction between all three NdAP3 proteins that may affect their expression.

### Searching for Genes Downstream of AP3 That Are Part of the Petal Identity and Development Networks

Because petals are completely suppressed in the homozygous *NdAP3-3* mutant, we assume that differentially expressed genes between the two homozygous *PP* and *pp* genotypes would include good candidates for petal identity and early development gene network in *N. damascena*. In *Arabidopsis*, AP3/PI heterodimer is bifunctional, acting either as activator or repressor of downstream target genes (Wuest et al., 2012). Interestingly, among the genes differentially expressed between the two floral morphs in *N. damascena*, we found more genes with a higher expression in the T morph than in the P morph, suggesting that NdAP3-3 and the complex it belongs to act more often as a repressor than as an activator at the early stages that we investigated.

Almost 50% of the morph DEGs could not be functionally annotated (Supplementary Table S3). Part of these transcripts could be non-coding sequences, or possibly misassembled sequences, a limitation of transcriptome assembly without a reference genome. Based on the results of homology searches in the genome of the closely related species *A. coerulea* and of experimental validation of a small set of unannotated sequences, we make the assumption that about 50% of these transcripts, possibly including non-coding transcripts, would be specific to *N. damascena* and could possibly be involved in species-specific processes. Annotation and GO-term assignment in the most differentially expressed gene set suggested a large involvement of nuclear activities, including regulation of transcription, and an enrichment in processes linked to cell proliferation (ribosome components, DNA or RNA binding, cell organization and biogenesis, and/or DNA or RNA or protein metabolism), which is consistent with the initiation of a novel organ. Most *Arabidopsis* homologous genes specific to or preferentially expressed in the *Nigella* petal at late developmental stages (Zhang et al., 2020) were found in our transcriptome. However, they were not differentially expressed between morphs at the early stages we investigated, supporting the hypothesis that different gene sets and networks operate during development to build the elaborate petal. Within the annotated set of DEGs between morphs, we found a homolog of *CRC*, a carpel developmental gene known to be repressed by B-function genes in early stages of floral development in *Arabidopsis* (Wuest et al., 2012). Consistent with this, we found that *NdCRC* was more expressed in the T morph than in the P morph at both stages. We found a few homologs of genes known to play a role in *Arabidopsis* petal organogenesis *sensu stricto* (Huang and Irish, 2016). A homolog of a DRNL transcription factor, one of the earliest markers of petal initiation, was found to be downregulated in the T morph, and to be a putative target of NdAP3-3 (Supplementary Table S7). Conversely, a homolog of KIP-RELATED PROTEIN 7, an inhibitor of petal initiation and cell proliferation, was found upregulated in the mutant. Although a homolog of *BIGPETAL* was found in the *Nigella* transcriptome, it was not differentially expressed between the two morphs. This low number of homologs in *N. damascena* may suggest a lack of functional conservation, but it may also be that the developmental stages investigated are not appropriate to observe an expression and/or an effect of NdAP3-3 on these genes. Indeed, it can be noted that a few of these genes are differentially expressed between the wild type and the *ap3.3* mutant in *Arabidopsis* at developmental stages that are similar to ours (Wuest et al., 2012).

To further investigate the conservation of the petal gene network, we took advantage of the knowledge of the AtAP3 gene network and the large amount of expression data available in *Arabidopsis*. Because the inactivation of *NdAP3-3* alone is sufficient to lose petal identity without affecting stamen identity in *N. damascena*, unlike the *AP3* mutation in *Arabidopsis*, we expected only partial conservation of the gene network between the two species. Among the most conserved set of genes between *Nigella* and *Arabidopsis* (11 NdH-BCG-DEG and 47 NdH-DEG, Supplementary Table S6) that are regulated by the *AP3* genes, almost half were homologs of direct targets of the AtAP3 protein, indicating an enrichment of AtAP3 target genes. Potential NdAP3-3 target genes included the three *NdAP3* paralogs, the *NdCRC* gene, as well as genes involved in hormone signaling and morphogenesis (Supplementary Table S7). Early repression of carpel developmental genes could be a conserved function of *AP3* genes (Wuest et al., 2012).

The percentage of target genes of AP3-3 homologous to AtAP3 target genes are in the same range in *A. coerulea* (Jiang et al., 2020) and *N. damascena* (Figure 4A). Combining these results, we found a set of 18 potential target genes that were conserved between the three species. Because the developmental stage investigated in *A. coerulea* was quite late, part of early target genes that could be conserved between species could have been missed. Nevertheless, our analyses point to a core set of conserved genes between three species belonging to two divergent clades of eudicots with independently derived petals (Carrive et al., 2020). These genes might encode conserved protein functions and be targeted by the common ancestor protein of the AP3-III and euAP3 lineages. Among these conserved genes, we found again the homologs of *AP3* and other transcription factors, as well as enzymes involved in cell wall biosynthesis (Supplementary Table S8). Interestingly, when comparing *Arabidopsis* and the two Ranunculaceae species, we noticed that different paralogs of a gene family could be recruited as the direct target of the AP3 proteins to fulfill a similar function (6 out 18, Supplementary Table S8). To confirm these conserved genes as part of the AP3 gene network at an early stage, both additional ChIP‐ and RNA-seq analyses should be carried out in *A. coerulea*, as well as ChIP-sequence experiments in *N. damascena* when the genome sequence will become available.

The comparative analysis of the *Nigella* differentially expressed genes and the putative target genes of AcAP3-3 revealed a higher conservation of the petal gene network between the two Ranunculaceae species than between them and *Arabidopsis* (Figure 4B). This is expected, because petals in Ranunculaceae have been evolved once independently from those of core eudicots (Carrive et al., 2020), and Ranunculaceae and core eudicots diverged around 130 million years ago (Magallón et al., 2015). Interestingly, among these conserved Ranunculaceae putative target genes, we found 40 genes that have *Arabidopsis* homologs with a differential expression between the wild type and the *ap3.3* mutant, but no AP3-binding sites in their regulatory regions, suggesting that they are not direct target genes of the AtAP3 protein. One hypothesis would be that these genes would have a flexible position in the petal gene network and could shift from an AP3 direct-to-indirect target position, depending on the species. New plant models for flower development chosen across the angiosperm phylogeny, and advanced DNA sequencing and analyses methods, should help addressing these hypotheses in the near future.

## Data Availability Statement

The datasets presented in this study can be found in online repositories. The names of the repository/repositories and accession number(s) can be found at: https://www.ncbi.nlm.nih.gov/geo/, GSE159429.

## Author Contributions

YD, NC, CD, DM, and SN designed the study. MG and HC performed the molecular work. LS-T, SB, and JC produced the RNA-seq data. VB, HB, JJ, and ED performed the bioinformatic work. YD, NC, DM, and CD performed the analyses. All authors contributed to the article and approved the submitted version.

### Conflict of Interest

The authors declare that the research was conducted in the absence of any commercial or financial relationships that could be construed as a potential conflict of interest.

## Abbreviations

AtAP3-BCG: 200 *Arabidopsis* genes whose expression is best correlated (100 positively and 100 negatively) with *AtAP3* expression
AtDEG: *Arabidopsis* genes whose expression differs between wild type and *ap3.3* mutant
AtBCG-DEG: *Arabidopsis* genes that are both AtDEG and AtAP3-BCG
DEG, differentially expressed genes
M_LFC: Log-fold change between the two morphs
NdH: *Nigella* genes homologous to AtDEG
NdH-BCG: *Nigella* genes homologous to AtAP3-BCG
NdH-BCG-DEG: NdH-BCG that are differentially expressed between the *Nigella* floral morphs
NdH-DEG: NdH that are differentially expressed between the *Nigella* floral morphs.
