# Supplementary material for: Transcriptome Analysis Reveals Putative Target Genes of APETALA3-3 During Early Floral Development in Nigella damascena L
Source: Front Plant Sci. 2021 Jun 4;12:660803. doi: 10.3389/fpls.2021.660803 (PMC8212990; doi:10.3389/fpls.2021.660803)
Supplement: Supplementary file 2 [file Presentation_1.pptx]

## Slide 1
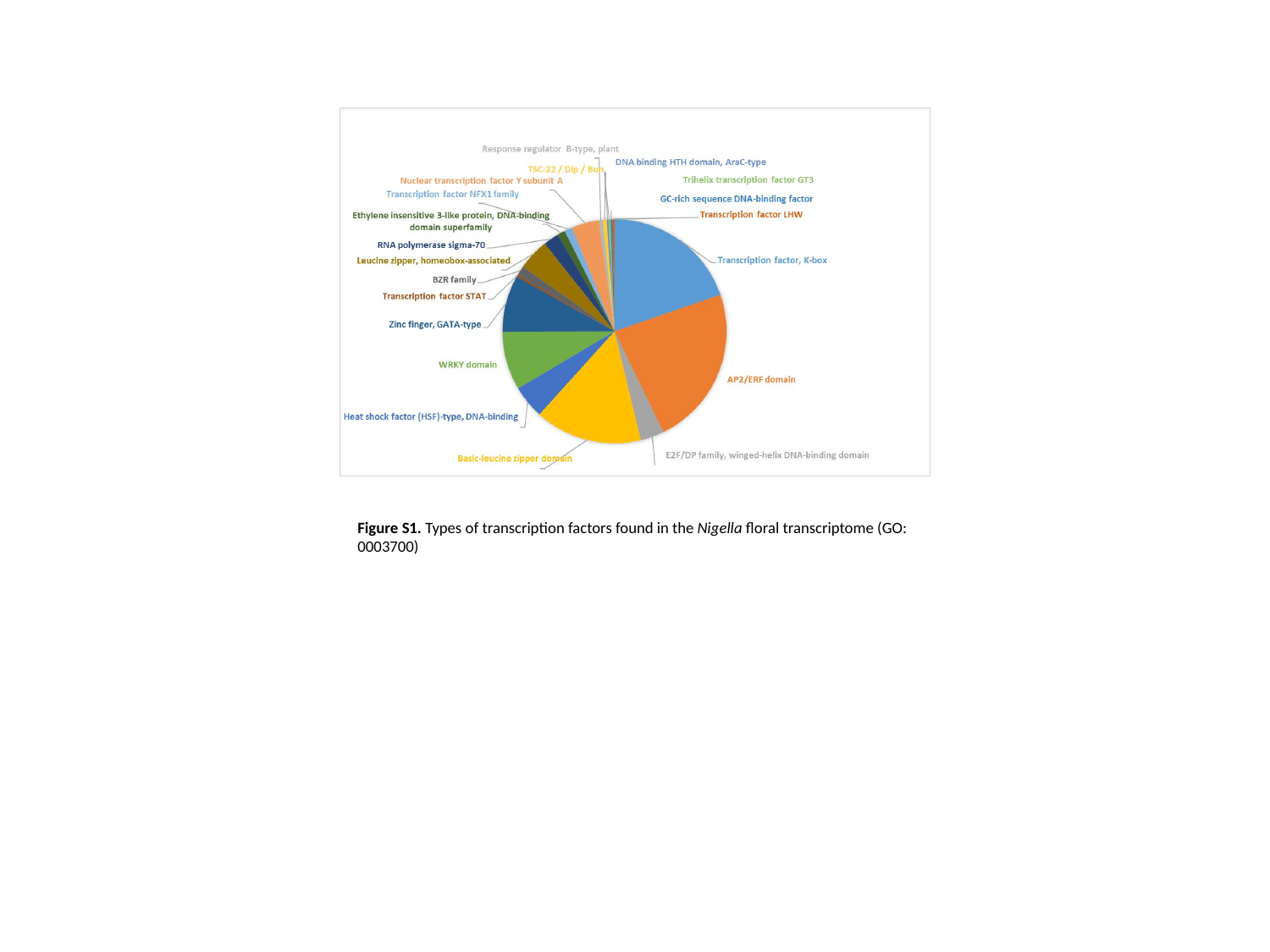

Figure S1. Types of transcription factors found in the Nigella floral transcriptome (GO: 0003700)

## Slide 2
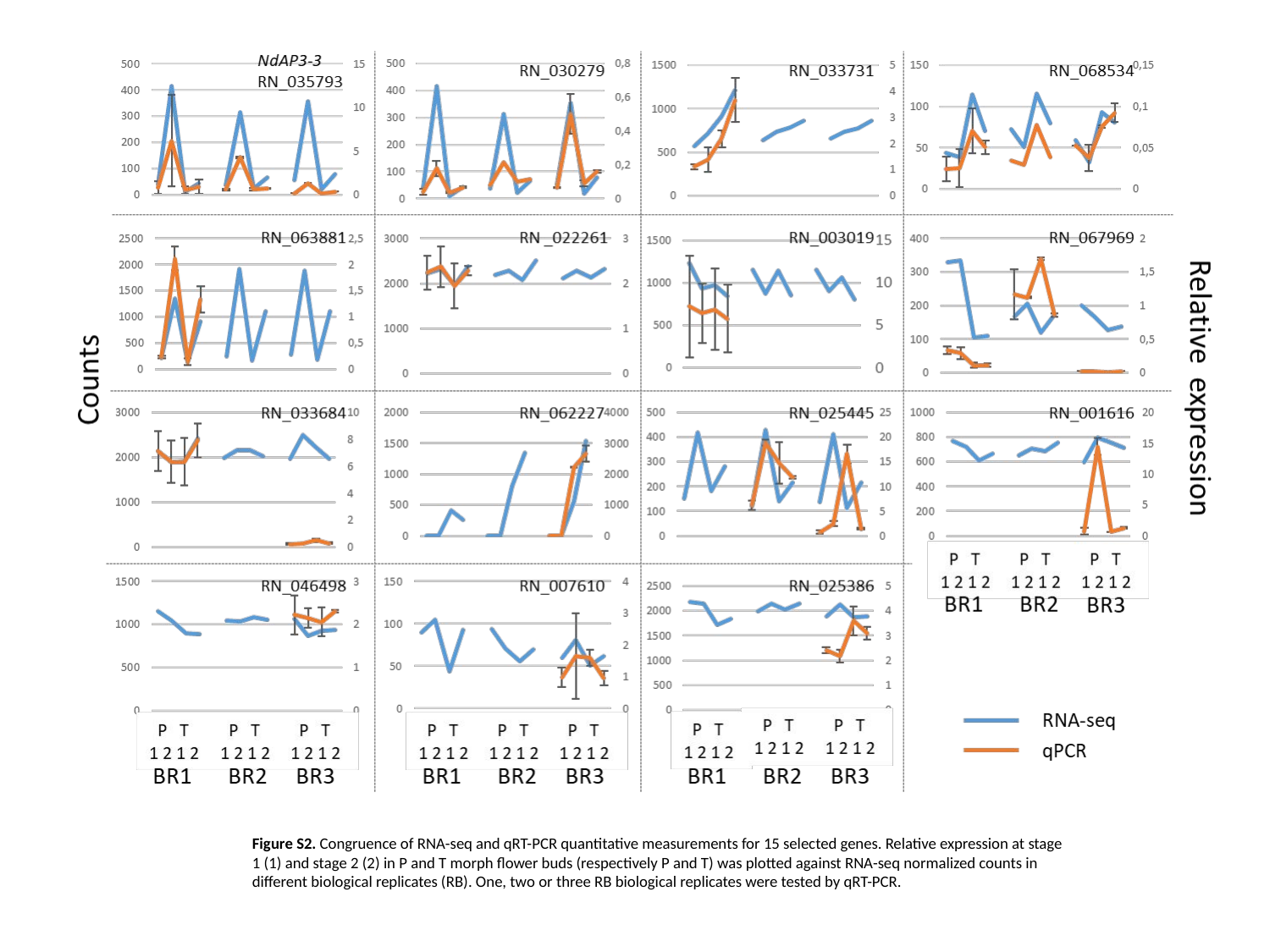

Figure S2. Congruence of RNA-seq and qRT-PCR quantitative measurements for 15 selected genes. Relative expression at stage 1 (1) and stage 2 (2) in P and T morph flower buds (respectively P and T) was plotted against RNA-seq normalized counts in different biological replicates (RB). One, two or three RB biological replicates were tested by qRT-PCR.

## Slide 3
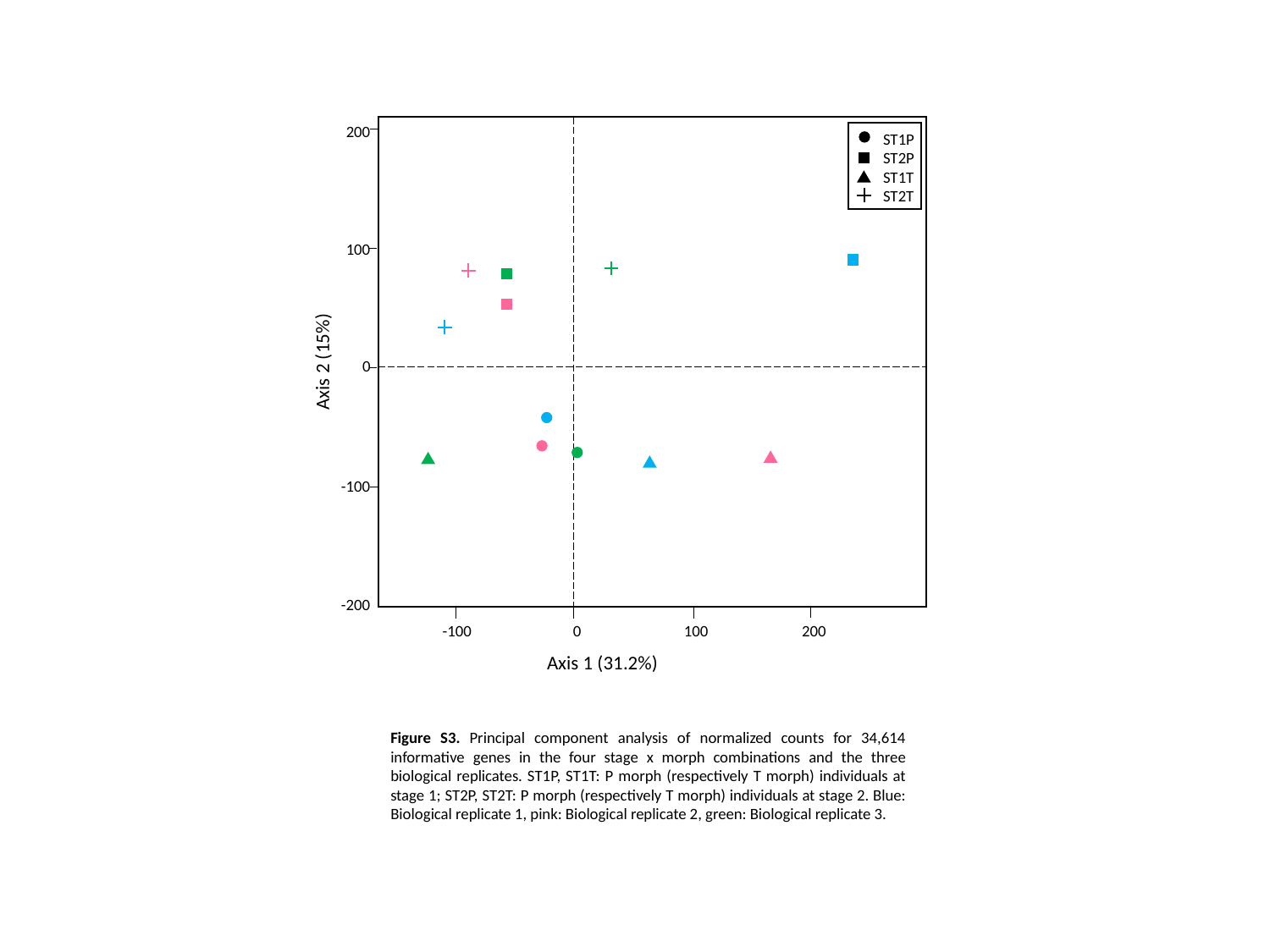

200
ST1P
ST2P
ST1T
ST2T
100
Dim2 (15%)
0
-100
-200
-100
0
100
200
Dim1 (31.2%)
Axis 2 (15%)
Axis 1 (31.2%)
Figure S3. Principal component analysis of normalized counts for 34,614 informative genes in the four stage x morph combinations and the three biological replicates. ST1P, ST1T: P morph (respectively T morph) individuals at stage 1; ST2P, ST2T: P morph (respectively T morph) individuals at stage 2. Blue: Biological replicate 1, pink: Biological replicate 2, green: Biological replicate 3.

## Slide 4
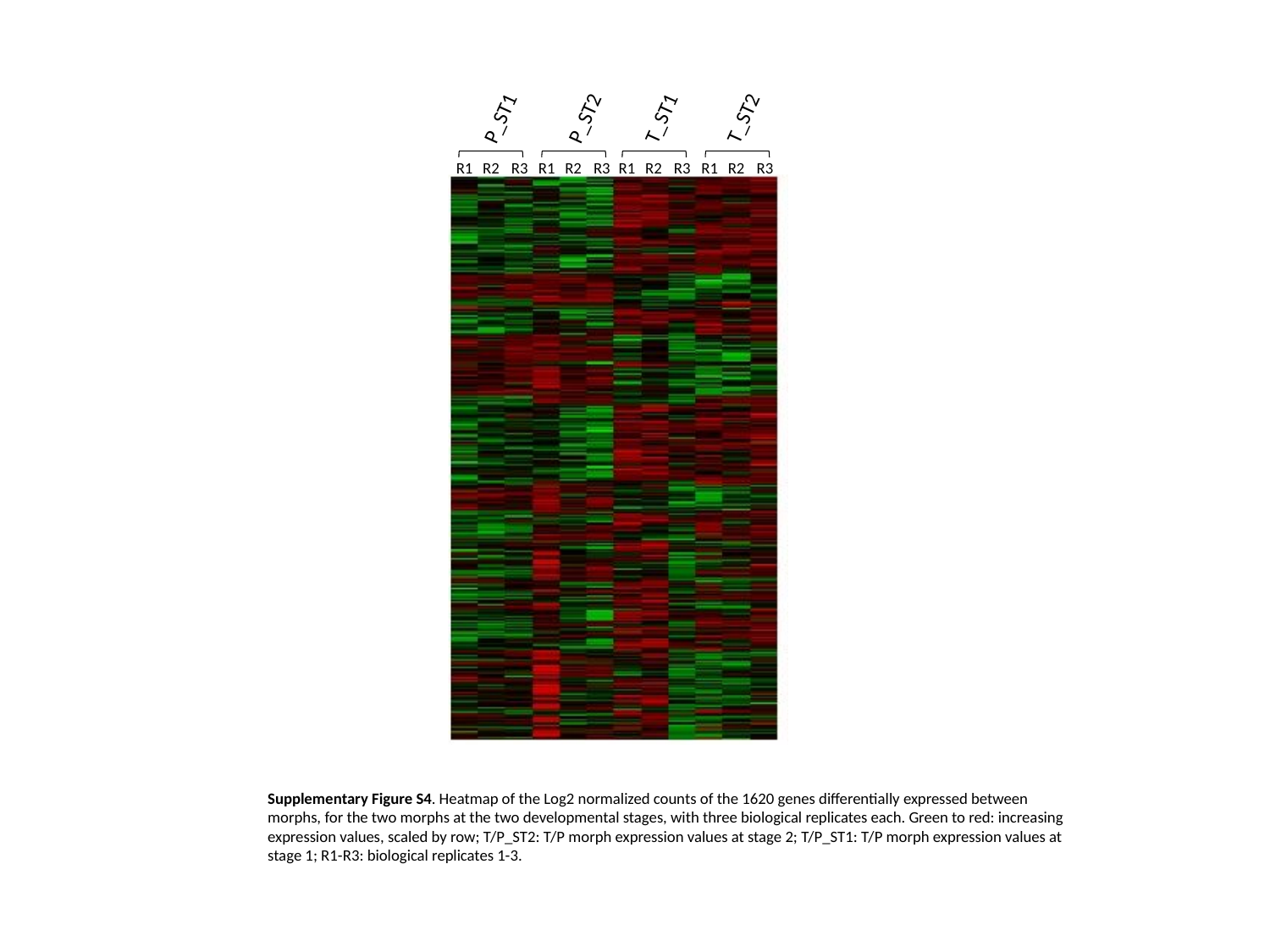

T_ST1
T_ST2
P_ST1
P_ST2
R1
R2
R3
R1
R2
R3
R1
R2
R3
R1
R2
R3
Supplementary Figure S4. Heatmap of the Log2 normalized counts of the 1620 genes differentially expressed between morphs, for the two morphs at the two developmental stages, with three biological replicates each. Green to red: increasing expression values, scaled by row; T/P_ST2: T/P morph expression values at stage 2; T/P_ST1: T/P morph expression values at stage 1; R1-R3: biological replicates 1-3.

## Slide 5
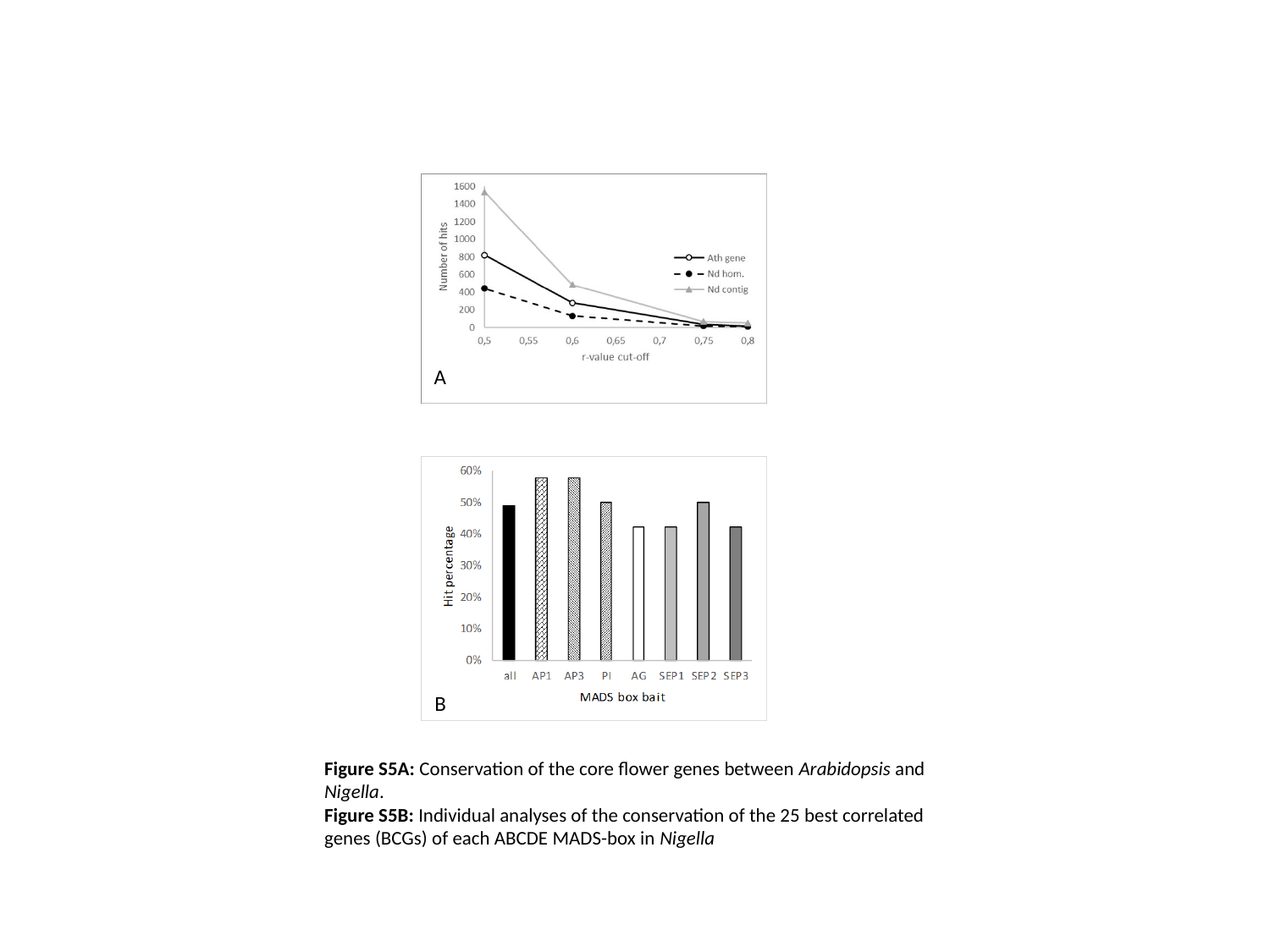

A
B
Figure S5A: Conservation of the core flower genes between Arabidopsis and Nigella.
Figure S5B: Individual analyses of the conservation of the 25 best correlated genes (BCGs) of each ABCDE MADS-box in Nigella
